# HMGB1: a key molecule linking chronic inflammation to complications in type 2 diabetes mellitus and a target for exercise intervention

**DOI:** 10.3389/fendo.2026.1769497

**Published:** 2026-02-27

**Authors:** Fu Pengyu, Xu Huiyun, Gong Lijing

**Affiliations:** 1 Department of Physical Education, Northwestern Polytechnical University, Xi’an, Shaanxi, China; 2 College of Life Sciences and Technology, Northwestern Polytechnical University, Xi’an, Shaanxi, China; 3 Key Laboratory of Exercise and Physical Fitness, Ministry of Education, Beijing Sport University, Beijing, China

**Keywords:** diabetic complications, exercise, HMGB1, inflammation, T2DM

## Abstract

The pathological process of type 2 diabetes mellitus (T2DM) is closely associated with chronic low−grade inflammation. High mobility group box 1 (HMGB1), a key damage−associated molecular pattern (DAMP), is frequently dysregulated in T2DM and is implicated in promoting insulin resistance (IR), β cell dysfunction, and the progression of multiple complications—including cardiovascular disease, nephropathy, cognitive impairment, myopathy, and dyslipidemia—primarily through activating signaling pathways such as RAGE/TLR4−NF−κB. Exercise, a cornerstone non−pharmacological intervention, effectively mitigates HMGB1−driven pathology through multifaceted mechanisms. These include direct downregulation of HMGB1 expression and suppression of its downstream inflammatory pathways, as well as indirect effects via improved glycemic control, enhancing autophagy, and reduced oxidative stress. This review aims to systematically examine the evidence for the role of HMGB1 in T2DM pathogenesis and its complications, and to evaluate exercise as a potential strategy to target this inflammatory pathway, thereby providing a theoretical framework for future therapeutic approaches.

Type 2 diabetes mellitus (T2DM) represents a profound global health crisis, driven not only by classical metabolic defects but also by a state of persistent, low-grade inflammation. While insulin resistance and β cell dysfunction are established pillars of T2DM pathogenesis (1), chronic inflammation is increasingly recognized as a key pathophysiological mechanism that appears to accelerates disease progression and underlies its systemic complications (2, 3). Within this inflammatory network, the damage-associated molecular pattern (DAMP) molecule high-mobility group box 1 (HMGB1) has been proposed as a key mediator. Normally a nuclear protein, HMGB1 can be released into the extracellular space during cellular stress or injury, where it activates receptors such as the receptor for advanced glycation end products (RAGE) and Toll-like receptors (TLRs), triggering potent pro-inflammatory signaling (e.g., nuclear factor kappa-B, NF-κB) and cytokine release (4). In T2DM, HMGB1 levels have been reported to correlate with insulin resistance, β cell impairment, and complications affecting the heart, kidney, nerves, and skeletal muscle (5, 6). Preclinical evidence thus positions HMGB1 as a promising therapeutic target; however, effective and clinically translatable means to modulate its activity remain underdeveloped (7, 8). Exercise is a cornerstone of T2DM management, with extensive evidence supporting its role in improving metabolic outcomes and reducing inflammation (9). Yet, the specific molecular pathways through which exercise exerts these benefits remain incompletely understood. A critical gap exists in understanding whether and how exercise influences the HMGB1 axis—a pathway strongly implicated in sustaining diabetic inflammation and tissue damage. Current evidence is fragmented, and a systematic analysis of exercise-mediated regulation of HMGB1 expression, release, and downstream signaling is lacking.

Therefore, this review aims to address this gap by: (1) synthesizing preclinical and emerging clinical evidence on the pathogenic role of HMGB1 across T2DM complications, and (2) critically examining the proposed mechanisms by which exercise directly and indirectly modulates the HMGB1 pathway. By integrating pre-clinical and clinical insights, we seek to provide a coherent mechanistic framework that not only clarifies how exercise improves T2DM outcomes but also potentially informs the development of targeted, HMGB1-based therapeutic strategies.

## 1 Biological characteristics of HMGB1

HMGB1 is a highly conserved nuclear protein whose dual roles—inside and outside the cell—underpin its significant importance in physiological and pathological contexts. With a molecular weight of approximately 25 kDa, HMGB1 comprises 215 amino acid residues. Its structure features two homologous DNA−binding domains, designated HMG−box A and HMG−box B, and a negatively charged acidic C−terminal tail. The A− and B−boxes constitute the functional core for DNA binding, while the C−terminal tail fine−tunes both DNA−binding affinity and protein−protein interactions (10). Under homeostatic conditions, HMGB1 is predominantly localized in the nucleus. Its canonical physiological functions encompass: (1) Nucleosome stabilization: by bending DNA to promote nucleosome assembly and structural integrity; and (2) Transcriptional regulation: serving as a co−factor that interacts with transcription factors such as p53 and nuclear factor kappa-B (NF−κB) to facilitate transcription−complex assembly, thereby contributing to the genetic regulation of cell proliferation, differentiation, and death (11).

HMGB1 shifts from a “nuclear housekeeper” to an “extracellular alarmin”, a transition central to its role in inflammation. This occurs via two main mechanisms. Passive release takes place during necrosis, injury, or severe irreversible stress, when nuclear membrane integrity is lost. Under these conditions, HMGB1—owing to its abundance and reduced DNA affinity—leaks from the nucleus into the cytoplasm and is ultimately released extracellularly, acting as a prototypical DAMP that alerts the immune system and initiates innate responses (12). Notably, in programmed cell death, HMGB1 remains chromatin−bound and is typically not released, highlighting the body’s ability to distinguish between death modalities (13). Active secretion, by contrast, is induced in immune cells such as monocytes/macrophages by stimuli like lipopolysaccharide (LPS), cytokines, or ROS (14). This regulated pathway involves post−translational modifications (e.g., acetylation) that promote HMGB1 translocation from the nucleus to the cytoplasm, followed by packaging into secretory vesicles (e.g., lysosomes) and release via exocytosis (15). Active secretion thus sustains immune−cell−driven inflammatory responses.

Once released extracellularly, HMGB1 elicits broad inflammatory responses and tissue damage primarily by engaging specific cell-surface receptors. The key receptors include RAGE—known for its pleiotropic ligand interactions—whose binding to HMGB1 is associated with mediating cellular chemotaxis, adhesion, and migration, and activates complex intracellular signaling cascades. HMGB1 also binds to TLR4, and this interaction, especially when HMGB1 forms complexes with molecules such as LPS, is known to potently amplifies inflammatory signaling.

The engagement of HMGB1 with its receptors triggers potent downstream signaling cascades, with the activation of the NF-κB pathway representing a central event. Specifically, HMGB1 binding to RAGE and/or TLR4 recruits and activates adaptor proteins and kinases, culminating in the phosphorylation and degradation of NF-κB inhibitors. This releases NF-κB (mainly the p65 subunit) from cytoplasmic retention, enabling its nuclear translocation. Inside the nucleus, NF-κB functions as a transcription factor that binds to promoters of multiple pro-inflammatory cytokine genes, driving their robust transcription and expression (16). These cytokines further amplify inflammation, creating a pro−inflammatory positive−feedback loop that contributes critically to insulin resistance and β cell dysfunction in T2DM. Importantly, the biological activity of HMGB1 is finely tuned by its redox state. Oxidation of specific cysteine residues generates distinct redox forms with markedly differing receptor−binding affinities and downstream effects, thereby adding considerable complexity to the HMGB1 signaling network (17).

## 2 Roles of HMGB1 in type 2 diabetes mellitus and its complications

HMGB1 critically contributes to the onset and progression of T2DM and its diverse complications by driving persistent inflammation, exacerbating insulin resistance, and triggering cellular injury, notably pyroptosis. Its impact extends across the entire spectrum of metabolic dysregulation and impinges upon multiple organ systems. It is important to note, however, that this mechanistic understanding is predominantly established in preclinical animal and *in vitro* models, highlighting the need for further validation in human clinical studies.

### 2.1 IR and β cell dysfunction

**Figure 1:**
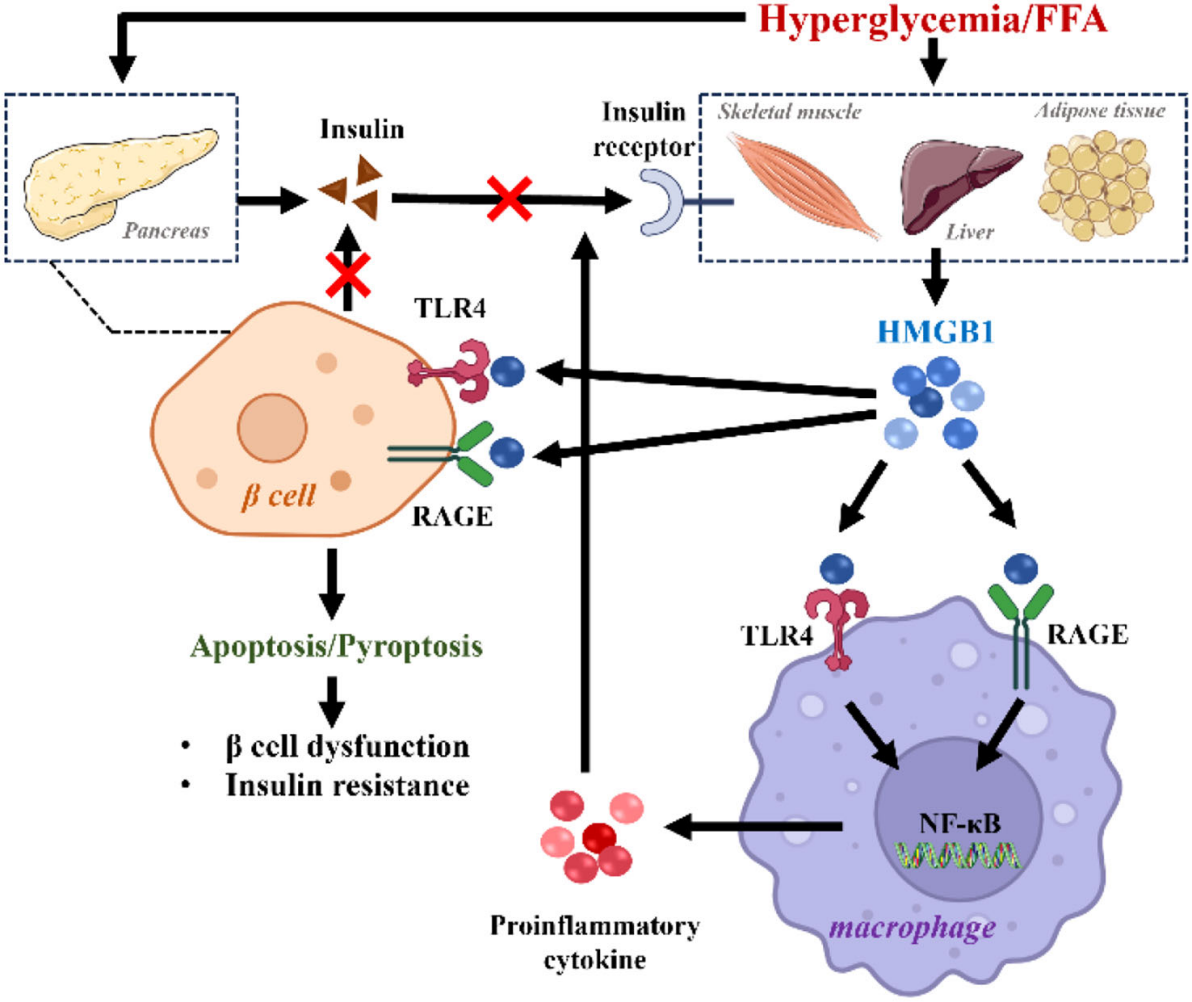
HMGB1 Regulation of Insulin Resistance and β Cell Dysfunction. Hyperglycemia and elevated FFA disrupt metabolite homeostasis, which in turn promotes the release of HMGB1. HMGB1 activates inflammatory pathways by engaging TLR4 and RAGE, thereby triggering the downstream NF−κB signaling cascade and driving macrophage-mediated inflammation. HMGB1, High-mobility group box 1; FFA, free fatty acids; TLR4, Toll-like receptor 4; RAGE, Receptor for advanced glycation end products; NF-κB, Nuclear factor-kappa B.

IR and pancreatic β cell dysfunction are central pathogenic components of T2DM, both of which appear to be adversely influenced by HMGB1 through its modulation of inflammatory signaling pathways (**Figure 1**) (18). Supporting this, HMGB1−knockout mice have shown enhanced insulin sensitivity and reduced hyperglycemia. In insulin−sensitive tissues—including adipose tissue, liver, and skeletal muscle—metabolic stressors such as hyperglycemia and elevated free fatty acids (FFA) stimulate HMGB1 release. Extracellular HMGB1 then binds to the cell−surface receptors RAGE or TLR4, potentially activating pro−inflammatory pathways such as NF−κB and c−Jun N−terminal kinase (JNK). This activation leads to aberrant serine/threonine phosphorylation of insulin receptor substrate (IRS), which disrupts normal tyrosine phosphorylation and is associated with impaired insulin signal transduction (19). Clinically, circulating HMGB1 levels correlate positively with the homeostatic model assessment of insulin resistance (HOMA-IR) in T2DM patients (18). Mechanistically, high glucose upregulates HMGB1 expression via the NF-κB pathway, a response associated with elevated pro-inflammatory cytokines and reversible by HMGB1 inhibition, suggesting a role for HMGB1-driven NF-κB signaling in promoting IR (20).

Within the islet microenvironment, chronic hyperglycemia and lipotoxicity synergistically promote a pro-inflammatory shift. Persistent metabolic stress induces the release of HMGB1 from β cells or infiltrating immune cells. Once released, HMGB1 engages TLR4 and RAGE on β cells, directly blunting glucose-stimulated insulin secretion while also triggering oxidative stress and inflammatory pathways that drive β cell apoptosis and pyroptosis. Importantly, necrotic β cells further release HMGB1, potentially creating a self-reinforcing vicious cycle that may contribute to progressive β cell failure (19).

### 2.2 Diabetic cardiovascular complications

Diabetic cardiovascular complications are the primary cause of death in patients with T2DM. HMGB1 serves as a key inflammatory mediator that has been implicated in actively promoting their development. Clinically, HMGB1 levels are closely linked to cardiovascular pathology. Patients with coronary artery disease or ST-segment elevation myocardial infarction have been reported to show significantly higher HMGB1 levels than healthy individuals, and atherosclerotic plaques contain increased numbers of HMGB1-producing macrophages, suggesting a role for HMGB1 in disease progression. Furthermore, T2DM patients with coronary heart disease also display elevated plasma HMGB1 (21). At the molecular level, HMGB1 is proposed to drive cardiovascular injury through several mechanisms. Within the vessel wall, HMGB1 acts as a potent inflammatory signal that activates endothelial cells, enhances monocyte adhesion and migration, stimulates vascular smooth muscle cell proliferation and migration, and promotes macrophage-to-foam-cell transformation—all of which collectively are thought to accelerate atherosclerotic plaque formation and progression (22).

HMGB1 directly contributes to myocardial injury and remodeling. In cardiac ischemia-reperfusion injury, HMGB1 inhibition reduces infarct size and diminishes levels of tissue injury markers, while exogenous HMGB1 aggravates damage. Under high-glucose conditions, HMGB1 released from cardiomyocytes and cardiac fibroblasts activates the RAGE/NF-κB pathway, which appears to drive inflammatory infiltration, fibrosis, and cardiomyocyte apoptosis in the heart. These changes may culminate in myocardial structural remodeling and functional impairment, characterized by diastolic and systolic dysfunction—the hallmark features of diabetic cardiomyopathy (23).

### 2.3 Diabetic nephropathy

Diabetic nephropathy (DN) is a prevalent and serious microvascular complication of T2DM. Emerging evidence indicates that serum HMGB1 levels could serve as a potential biomarker for the early detection of DN (24). HMGB1 plays a central pathogenic role in DN progression. Hyperglycemia stimulates resident renal cells—including mesangial cells, podocytes, and renal tubular epithelial cells—to express and secrete HMGB1 into the extracellular milieu. There, HMGB1 engages membrane receptors, mainly RAGE and TLRs, initiating innate immune responses marked by sustained NF-κB pathway activation. This cascade drives abundant local pro-inflammatory cytokine production, promoting monocyte/macrophage infiltration and accumulation within renal tissue, which amplifies renal inflammation. The ensuing HMGB1-mediated inflammatory response further triggers podocyte autophagy, apoptosis, and epithelial-mesenchymal transition, alongside excessive extracellular matrix deposition. It has been proposed that under chronic hyperglycemia in DN, HMGB1 release establishes a pathogenic positive-feedback loop that continuously upregulates pro-fibrotic factors, ultimately culminating in glomerulosclerosis and tubulointerstitial fibrosis, and driving progressive renal function decline (25).

Furthermore, HMGB1 modulates cell death-related pathways, thereby critically shaping the course of DN. Recent findings highlight that in renal tubular epithelial cells exhibiting high ferroptosis sensitivity, suppression of HMGB1 secretion and modulation of the HMGB1/nuclear factor (erythroid-derived 2)-like 2 (Nrf2) signaling axis under hyperglycemic conditions may constitute a key mechanism mitigating diabetic kidney injury (26).

### 2.4 Diabetic cognitive dysfunction

T2DM is a major risk factor for Alzheimer’s disease and vascular dementia, though the mechanisms underlying this link are complex. Studies show that hyperglycemia not only drives neurodegeneration directly but may also promote dementia through disruption of the blood−brain barrier. Within this pathological cascade, HMGB1−mediated neuroinflammation has been proposed to act as a central bridge connecting metabolic dysfunction to brain damage.

HMGB1 appears to promote cognitive impairment through interconnected mechanisms. Hyperglycemia and cerebral insulin resistance first activate microglia, triggering HMGB1 release. Extracellular HMGB1 then further stimulates microglia via the TLR4/NF-κB pathway, which may create a pro-inflammatory feedback loop and promoting nucleotide-binding oligomerization domain-like receptor protein 3 (NLRP3) inflammasome assembly. NLRP3 activation cleaves cysteine-dependent aspartate-specific proteases-1 (Caspase-1), leading to Interleukin-1β (IL-1β) release and possible vascular pyroptosis, which exacerbates neuroinflammation and disrupts blood-brain barrier integrity (27). Furthermore, in the hippocampus—a key region for learning and memory—this HMGB1-driven inflammation impairs synaptic plasticity and promotes neuronal degeneration and death, which may ultimately contribute to cognitive dysfunction and memory loss (28). Importantly, therapeutic targeting of this pathway shows promise: inhibition of the NLRP3 inflammasome downstream of HMGB1, for example with the selective inhibitor MCC950, attenuates blood-brain barrier damage, reduces inflammation and cell death, and improves barrier integrity in experimental models, supporting the potential central role of the HMGB1-NLRP3 axis in diabetic encephalopathy (29).

### 2.5 Diabetic muscle atrophy

Skeletal muscle serves as the major organ for glucose disposal and storage. Diabetic myopathy, marked by progressive skeletal muscle wasting and dysfunction, worsens overall glycemic control. In T2DM, systemic inflammation and insulin resistance upregulate HMGB1 expression in skeletal muscle. While direct mechanistic studies of HMGB1 in T2DM-associated myopathy are still limited, available evidence positions HMGB1 as a potential key molecular target in this pathological process.

Hyperglycemia-induced HMGB1 upregulation may initiate inflammation-driven pyroptosis and is thought to contribute to pathological muscle remodeling (30). Uncontrolled hyperglycemia also promotes the irreversible, non−enzymatic formation of advanced glycation end products (AGEs). The accumulation of AGEs in skeletal muscle allows binding to the receptor RAGE, which can trigger intracellular events—such as inflammatory signaling and excessive ROS production—that are implicated in driving muscle atrophy. Downstream of HMGB1, activation of the NLRP3 inflammasome, a key complex that processes pro−IL−1β into its active form, depends on TLR4 signaling. Active IL−1β may then engage pathways including mitogen-activated protein kinase (MAPK) and NF−κB, which contribute to both systemic insulin resistance and skeletal muscle wasting. Furthermore, circulating LPS can act as a TLR4 agonist, potentially activating MAPK and NF−κB pathways independent of cytokines, thereby potentially contributing to muscle atrophy (31).

### 2.6 Diabetic dyslipidemia

Dyslipidemia is a hallmark of T2DM, and HMGB1 appears to contribute centrally to obesity-related metabolic dysregulation, potentially engaging in a mutually reinforcing pathogenic cycle. (1) HMGB1 may function as an adipocytokine whose expression is modulated by adipose tissue status: its levels are reduced in obese adipose depots, yet it is passively released from necrotic adipocytes and actively secreted under cellular stress or hypoxia. HMGB1 signals through RAGE and TLR4 in a manner influenced by its redox state and post-translational modifications, thereby potentially affecting IR (32). (2) Extracellular HMGB1 binds RAGE/TLR4 in adipose tissue, which is thought to initiate a self−sustaining inflammatory loop that recruits macrophages and drives their polarization toward a pro−inflammatory M1 phenotype. These activated macrophages release tumor necrosis factor-alpha (TNF−α), IL−1β, and additional HMGB1, thereby helping to sustain chronic adipose tissue inflammation (33). Functionally, exogenous HMGB1 has been shown in experimental settings to inhibit lipolysis in mature adipocytes, directly disrupting lipid metabolism. (3) HMGB1-driven inflammation is proposed to systemically perturbs lipid homeostasis by enhancing hepatic *de novo* lipogenesis, inhibiting fatty acid β-oxidation, and promoting adipose tissue lipolysis, which elevates circulating free fatty acids (FFA) and exacerbates hypertriglyceridemia and hepatic steatosis. These lipotoxic mediators, including FFA and oxidized low-density lipoprotein (ox-LDL), in turn stimulate further HMGB1 release from immune and parenchymal cells, amplifying inflammation and insulin resistance in a self-propagating vicious cycle (34).

**Figure 2:**
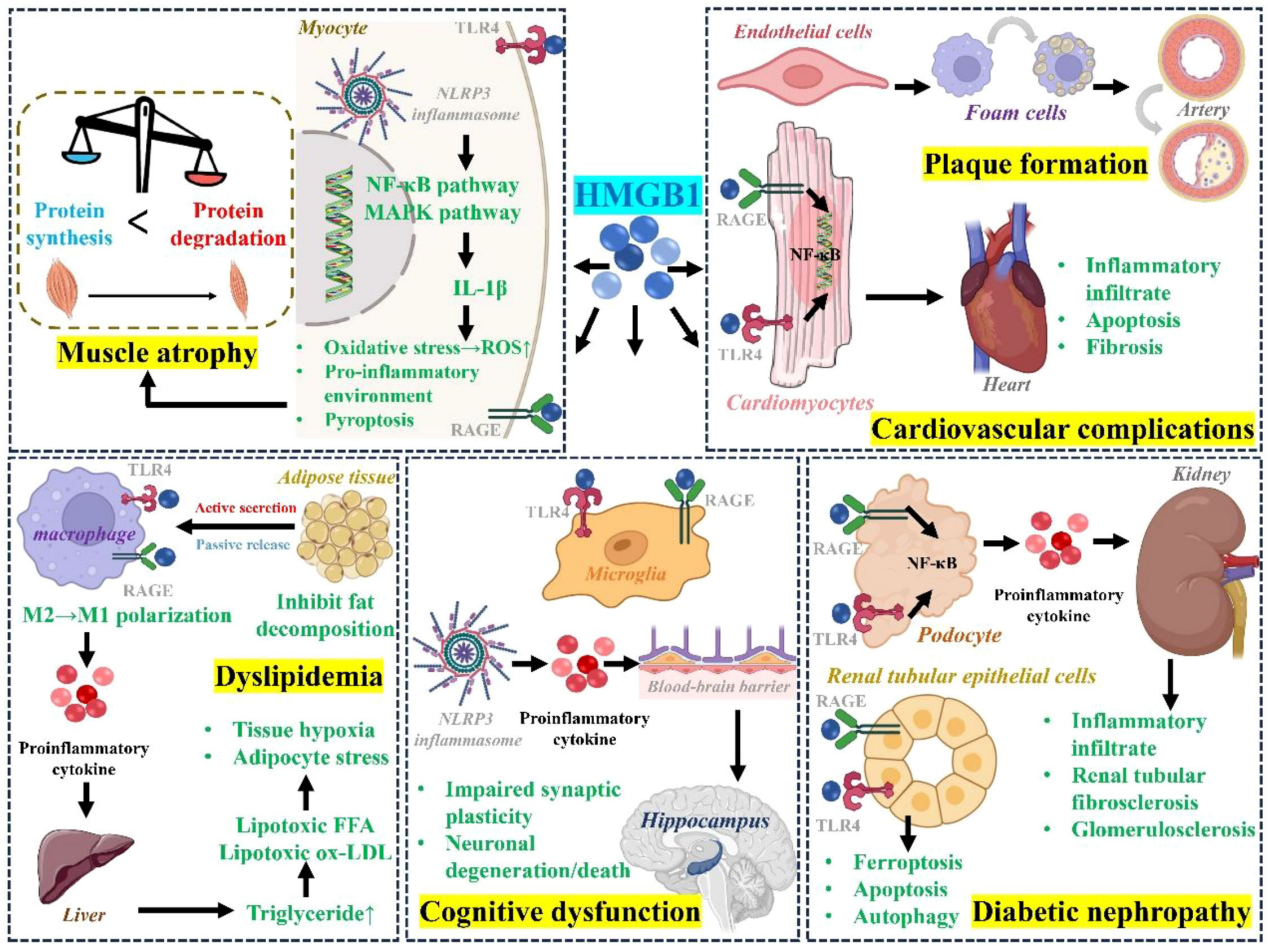
Systemic inflammatory network driven by HMGB1 in T2DM complications. Metabolic disturbances promote the release of HMGB1, which activates inflammatory signaling predominantly through TLR4 and RAGE receptors. This triggers downstream NF−κB pathways, leading to oxidative stress, cytokine production, and a pro-inflammatory environment. Consequently, this cascade contributes to multi-organ injury, including myocyte atrophy, vascular inflammation, cardiac fibrosis, adipocyte stress, renal/hepatic damage, and neuroinflammation via microglial activation, collectively linking metabolic stress to systemic complications. HMGB1, High-mobility group box 1; FFA, free fatty acids; TLR4, Toll-like receptor 4; RAGE, Receptor for advanced glycation end products; NF-κB, Nuclear factor-kappa B; NLRP3, Nucleotide-binding oligomerization domain-like receptor protein 3.

The proposed involvement of HMGB1 in the regulatory mechanisms underlying these diabetic complications is illustrated in **Figure 2**.

## 3 Exercise−mediated regulatory mechanisms of HMGB1 in T2DM

Physical inactivity is a significant risk factor for T2DM onset and progression. Research demonstrates that chronic sedentary behavior promotes macrophage infiltration in skeletal muscle and impairs insulin sensitivity (35). Conversely, regular physical exercise represents a cornerstone strategy for mitigating systemic low−grade inflammation.

### 3.1 Direct effects of exercise on HMGB1 expression

As an important alarmin, HMGB1 is integral to exercise-mediated immune and metabolic adaptation. While acute exercise temporarily increases circulating HMGB1 to initiate an adaptive inflammatory response, long-term regular training consistently lowers circulating HMGB1 levels in T2DM (36, 37). **Table 1** summarizes the contrasting regulatory effects of two exercise modalities on the organism.

**Table 1:** Contrasting effects of acute exercise *vs.* chronic exercise training on HMGB1 dynamics and their physiological implications.

Feature	Acute (especially vigorous) exercise	Chronic (regular) exercise training
HMGB1 Level	Transient increase in circulation and active tissues (38).	Sustained reduction in baseline levels across key tissues (39).
Primary Implication	Alarmin/”Danger” Signal: Initiates a controlled, adaptive inflammatory response to prepare for repair and adaptation (40).	Resolution & Adaptation: Contributes to long-term anti-inflammatory homeostasis and metabolic improvement (41).
Proposed Key Mechanisms	1. Cellular stress (mechanical, metabolic, oxidative) triggers passive release from muscles or active secretion from immune cells. 2. Temporary inhibition of autophagy may reduce clearance.	1. Improved metabolic milieu (glycemia, insulin sensitivity) removes chronic stimuli for HMGB1 expression. 2. Enhanced antioxidant defenses and autophagy promote clearance of HMGB1 and its inducers. 3. Epigenetic and transcriptional downregulation of HMGB1 and its receptors.
Major Signaling Pathways Involved	TLR4/MyD88, RAGE, redox-sensitive pathways.	RAGE/NF-κB downregulation, AMPK/PGC-1α activation, Nrf2/ARE antioxidant response.
Net Effect in T2DM Context	A necessary, transient provocation that may appear contradictory but is part of normal exercise physiology. Poor recovery could prolong inflammation (42).	Therapeutic foundation. Underlies the chronic anti-inflammatory, organ-protective, and insulin-sensitizing benefits of exercise (20).

Regular aerobic exercise effectively normalizes the elevated HMGB1 expression observed in T2DM models. For example, endurance running has been shown to significantly reduces both HMGB1 protein and mRNA levels in the hippocampus, liver, and adipose tissue of experimental models. This suppression is thought to result from exercise-induced improvements in local energy metabolism and oxidative stress, which diminish the triggers for HMGB1 release. Within the cardiovascular system, exercise alleviates hyperglycemia and hemodynamic stress, reducing injury to cardiomyocytes and the vascular endothelium and thereby limiting HMGB1 leakage and secretion.

At the molecular level, exercise−induced shifts in the cellular redox balance can modify the oxidation state of key residues in HMGB1, thereby influencing its post−translational modifications. Moderate exercise−associated oxidative stress may drive HMGB1 toward distinct redox isoforms with altered immunomodulatory activities—including a shift to a chemotactic conformation—thus potentially fine−tuning its inflammatory functions. Moreover, exercise appears to regulate where HMGB1 is located inside muscle cells and how it interacts with proteins involved in cellular “clean-up” (autophagy). In particular, eccentric (lengthening) exercise has been shown in preclinical studies to promote a process in which HMGB1 helps initiate autophagy—a key mechanism for removing damaged cellular components—in skeletal muscle (43). Additionally, acute exercise can modulate HMGB1−dependent crosstalk between macrophages and muscle cells (40), suggesting its potential role in exercise−elicited immunometabolic communication.

The regulatory effect of exercise on HMGB1 appears to differ across modalities, though current evidence is still preliminary. For example, one study in individuals with insulin resistance found that high-intensity interval training (HIIT) significantly reduced circulating HMGB1 and related pro-inflammatory cytokines (39), suggesting that exercise intensity or mode may selectively influence the HMGB1 pathway. However, comparative studies among HIIT, aerobic exercise, and resistance training are required to verify these modality-specific effects and to establish optimal exercise prescriptions for HMGB1 modulation. In streptozotocin/high−fat diet (STZ/HFD) -induced T2DM mice, aerobic exercise improves glycemic control and insulin sensitivity partly through suppression of hepatic HMGB1 expression. Notably, liver-specific knockout of HMGB1 attenuates the metabolic benefits of exercise (20), supporting the notion that hepatic HMGB1 as a key target mediating exercise-induced regulation of glucose metabolism.

### 3.2 Regulation of HMGB1 downstream signaling pathways by exercise

Exercise not only diminishes HMGB1 release but also appears to potently inhibits its downstream signaling (40). Research indicates that exercise downregulates RAGE expression in multiple tissues—including skeletal muscle, liver, and aorta—which may limit HMGB1 receptor binding and thereby help suppress downstream pathway activation (44). As a result, NF-κB signaling tends to be attenuated, evidenced by decreased inhibitor of nuclear factor kappa B alpha (IκBα) degradation and reduced nuclear translocation of the NF-κB p65 subunit. This in turn leads to a marked decline in the transcription and secretion of pro-inflammatory cytokines such as TNF-α and IL-6, ultimately helping to mitigate systemic and tissue-specific inflammation (45).

In the hippocampus of T2DM mice, exercise attenuates microglial overactivation (46), in part by blocking the HMGB1-TLR4 axis, thereby inhibiting NLRP3 inflammasome assembly and activation. Since the NLRP3 inflammasome plays a key role in IL-1β maturation and pyroptosis, its suppression through exercise significantly lowers hippocampal levels of activated caspase-1 and mature IL-1β. This reduction alleviates neuroinflammation and limits neuronal injury (47), offering a plausible molecular basis for the ability of exercise to improve cognitive impairment associated with diabetes.

### 3.3 Indirect regulation of HMGB1 through non-HMGB1-dependent pathways by exercise

**Figure 3:**
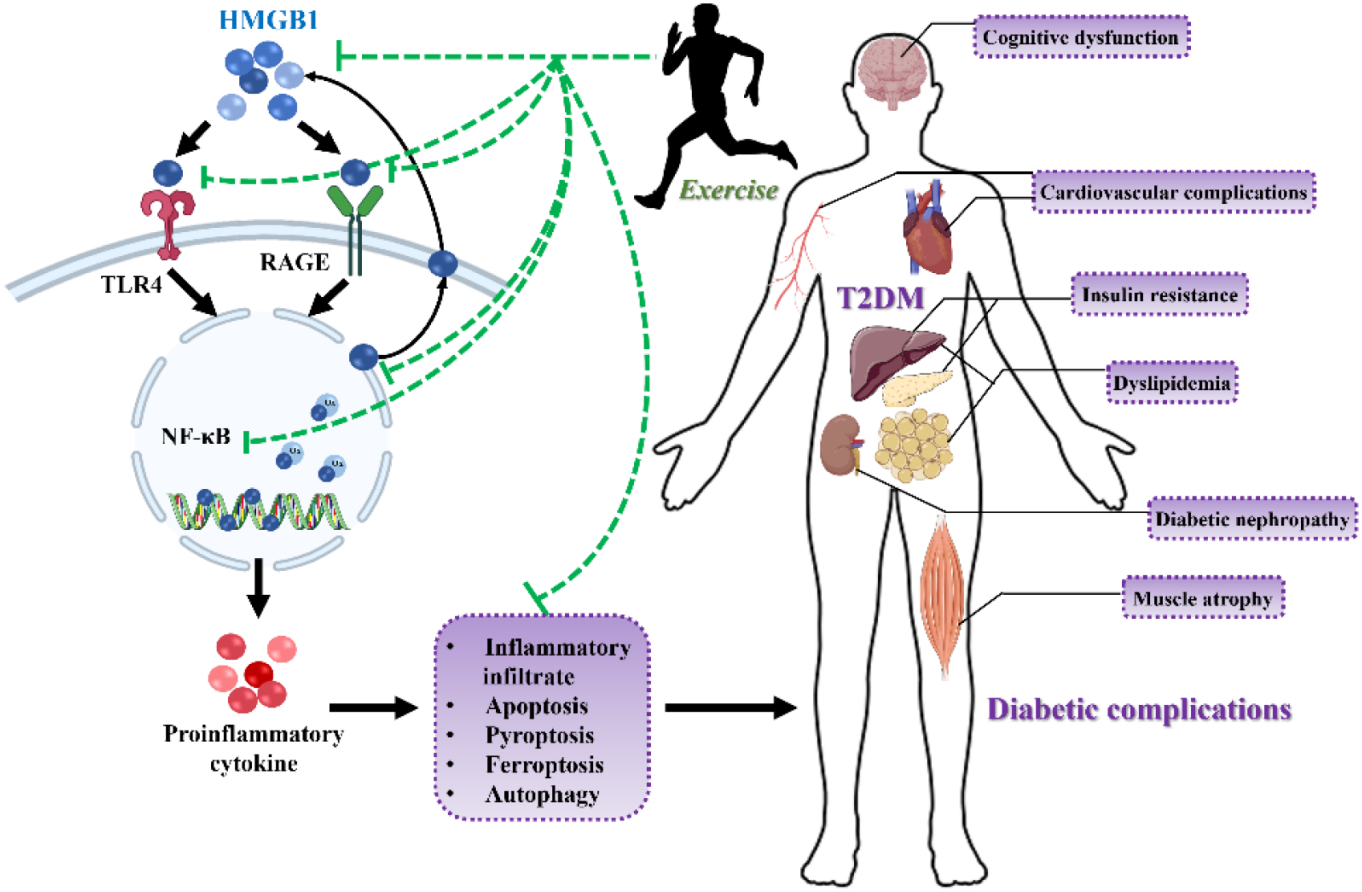
Proposed role of HMGB1 in the pathogenesis of T2DM complications. The diagram illustrates how the activation of the HMGB1/NF-κB inflammatory axis may contribute to the progression of T2DM and its systemic complications. This pathway promotes inflammatory cell infiltration and drives various modes of regulated cell death, including apoptosis, pyroptosis, ferroptosis, and altered autophagy. These cellular events collectively lead to tissue damage and dysfunction across multiple organ systems, manifesting as cognitive impairment, cardiovascular disease, insulin resistance, dyslipidemia, diabetic nephropathy, and muscle atrophy. T2DM, Type 2 diabetes mellitus; HMGB1, High-mobility group box 1; FFA, free fatty acids; TLR4, Toll-like receptor 4; RAGE, Receptor for advanced glycation end products; NF-κB, Nuclear factor-kappa B.

Beyond directly modulating HMGB1, exercise may counteract HMGB1 pathogenicity by ameliorating the systemic metabolic milieu. First, it lowers “glucotoxicity” by promoting skeletal muscle glucose uptake and inhibiting hepatic gluconeogenesis (48). Because hyperglycemia potently induces HMGB1 expression (49), this metabolic improvement likely contributes to the indirect suppresses HMGB1. Second, exercise activates autophagic flux in tissues such as the hippocampus and skeletal muscle of T2DM mice (50). Enhanced autophagy is thought to alleviate HMGB1−linked inflammation by clearing inflammatory mediators (e.g., HMGB1 and NLRP3 components) and removing damaged mitochondria to reduce ROS (51), with autophagic activation correlating with lower HMGB1 levels and better cognitive outcomes (52). Third, exercise boosts endogenous antioxidant defenses, limiting ROS accumulation (53); since oxidative stress triggers HMGB1 release (54), this antioxidant effect helps restrain HMGB1 activation and can disrupt the oxidative−inflammatory cycle. Furthermore, exercise help curb Caspase−1−dependent pyroptosis by inhibiting the TLR4/NLRP3 axis (55), while improved metabolism and autophagy may help eliminate pyroptotic triggers (56). Given the close tie between HMGB1 and pyroptosis, attenuated pyroptosis reduces cell rupture and inflammatory release (57), thereby potentially forming an anti−inflammatory feedback loop that could help break the self−perpetuating inflammation in T2DM. Possible mechanisms by which long−term exercise modulates HMGB1 to ameliorate T2DM and its complications are illustrated in **Figure 3**.

### 3.4 HMGB1 as a potential biomarker for exercise intervention

Based on its dynamic regulation by exercise, HMGB1 may be positioned as a promising translational biomarker for guiding and monitoring T2DM management. First, circulating HMGB1 levels could serve as an objective molecular readout to assess the anti-inflammatory efficacy of different exercise prescriptions, complementing traditional metabolic metrics like HbA1c. Second, given its implicated role in early-stage complications, tracking exercise-induced changes in HMGB1 may help identify responders versus non-responders to lifestyle intervention, potentially paving the way for personalized strategies. However, key challenges must be addressed before clinical translation: standardized assays for detecting specific HMGB1 isoforms are needed; longitudinal studies are required to define the temporal dynamics and prognostic value of HMGB1 changes post-exercise; and its tissue-specific release patterns necessitate careful interpretation of systemic levels. Future exercise trials that incorporate serial HMGB1 measurements alongside deep phenotyping would be crucial to validate its utility as a theragnostic biomarker, ultimately helping to bridge mechanistic insights with individualized patient care.

## 4 Conclusions and future prospects

This review systematically summarizes the central role of HMGB1 as a key inflammatory mediator in T2DM and its complications, and details how exercise modulates the HMGB1 pathway through both direct and indirect mechanisms, thereby conferring potential anti−inflammatory, metabolic, and organ−protective benefits. However, current evidence remains limited, as most findings derive from animal and *in vitro* studies, with a paucity of clinical data on how exercise influences HMGB1 in T2DM patients; moreover, no consensus exists on the optimal exercise regimen for regulating HMGB1, and the tissue−specific roles of HMGB1 in mediating exercise effects as well as its network−based regulatory mechanisms require deeper investigation. To address these gaps, future research should prioritize several concrete directions: first, conducting well−designed randomized controlled trials (RCTs) in T2DM cohorts to compare different exercise modalities while measuring HMGB1 levels, its receptors, and downstream cytokines to map pathway responses; second, performing translational studies to investigate whether exercise influences the relative abundance of distinct HMGB1 redox isoforms and how these changes correlate with metabolic and inflammatory outcomes; third, exploring the synergistic potential of combining exercise with pharmacological agents that target the HMGB1 pathway as a multi−modal therapeutic strategy; and fourth, building on this evidence to develop precise, personalized exercise prescriptions aimed at optimizing HMGB1 pathway modulation for different complication phenotypes. By pursuing these directions, the field can work toward solidify HMGB1’s role as both a therapeutic target and a biomarker for monitoring exercise efficacy, ultimately helping to translate mechanistic insights into improved clinical strategies for T2DM.
